# Do Provider Characteristics and Perceptions Influence the Adoption and Implementation of the Transdiagnostic Intervention for Sleep and Circadian Dysfunction (TSC) in Community Mental Health Centers?

**DOI:** 10.1007/s10597-025-01552-y

**Published:** 2025-10-31

**Authors:** Marlen Diaz, Anne E. Milner, Laurel D. Sarfan, Emma R. Agnew, Sophia M. Oliver, Kiely Bol, Allison G. Harvey

**Affiliations:** 1 University of California, Berkeley, Berkeley, CA USA; 2 Department of Research and Evaluation, Kaiser Permanente Southern California, Pasadena, CA USA

**Keywords:** Implementation outcomes, Provider characteristics, Provider perceptions, Community mental health, Transdiagnostic and modular treatments

## Abstract

Understanding the factors that influence the adoption and implementation of evidence-based practices (EBPs) is crucial for expanding access to high-quality treatments for serious mental illness (SMI). This study evaluated how provider characteristics and perceptions influence adoption and implementation outcomes of the Transdiagnostic Intervention for Sleep and Circadian Dysfunction (TSC) in community mental health centers (CMHCs). CMHC providers (*N* = 124) were trained to deliver TSC to CMHC patients diagnosed with SMI (*N* = 395). Providers completed measures on demographic and professional characteristics. Additionally, assessments conducted after providers completed TSC training and delivered TSC to a patient measured provider perceptions of TSC (i.e., treatment fit, willingness and confidence) and adoption and implementation outcomes (i.e., referrals, sessions delivered, and treatment completion). Providers reported highly positive perceptions of TSC. Providers’ education was associated with number of referrals and treatment completion. Additionally, provider perceptions of the TSC’s appropriateness and feasibility at post-training predicted later perception of treatment fit. Overall, findings suggest providers perceive TSC to be well-suited for CMHCs, where provider characteristics are diverse, which bodes well for sustainment and scalability in community settings.

**Clinical Trial Registration**: This study was preregistered on clinicaltrials.gov (identifier: NCT04154631) on November 6, 2019. https://clinicaltrials.gov/study/NCT04154631.

## Introduction

Implementing effective innovations is critical for expanding public access to high-quality treatments for serious mental illness (SMI) (Wang et al., 2002). However, one key factor researchers often overlook in the adoption and implementation of evidence-based practices (EBPs) is the characteristics of the providers delivering them. Provider characteristics include demographic factors such as type of education/degree, amount of professional experience (e.g., years and/or caseload), training (e.g., state licensure status), and primary discipline/theoretical orientation (Aarons et al., 2011). Dissemination and implementation frameworks highlight the important influence that provider characteristics likely have on adoption and implementation outcomes. For example, the Exploration, Preparation, Implementation, Sustainment framework (EPIS; Aarons et al., 2011), the Consolidated Framework for Implementation Research (CFIR; Damschroder et al., 2022), and the Reach, Effectiveness, Adoption, Implementation, and Maintenance framework (RE-AIM; Glasgow et al., 1999) all identify provider level characteristics as important determinants of the adoption and implementation of EBPs by individuals and agencies. A systematic review, however, points out that individual provider characteristics are underreported in empirical and evaluation articles, particularly because researchers find it difficult to collect this information (Gaglio et al., 2013). Thus, more research is needed to understand how provider characteristics influence adoption and implementation outcomes to increase access to EBPs.

Although limited, prior research has evaluated the association between provider characteristics and one type of implementation outcome—namely provider *perceptions* of various EBPs. The latter is important because holding a positive perception of EBPs has been associated with other downstream implementation outcomes, such as the adoption of EBPs (e.g., Gallo & Barlow, 2012; Nelson & Steele, 2007). For instance, researchers have suggested that provider characteristics including a higher level of education (e.g., Aarons, 2004) or fewer years of professional experience (e.g., Aarons, 2004; Ametaj et al., 2021; Barnett et al., 2017) are associated with more favorable perceptions of EBPs. However, other studies have found no such association (e.g., Barnett et al., 2017; Brookman-Frazee et al., 2009; Burgess et al., 2016; Louie et al., 2022). Another line of research shows that providers with higher caseloads tend to have poorer perceptions and are less willing to adopt EBPs (e.g., Barnett et al., 2017; Brennan et al., 2022; Nelson et al., 2006). Yet, there are mixed findings regarding the role of providers’ state licensure status and theoretical orientation in influencing perceptions of EBPs (e.g., Aarons, 2004; Barnett et al., 2017; Nakamura et al., 2011; Nelson & Steele, 2007). Taken together, these studies suggest that the influence of provider characteristics on perceptions of EBPs remains unclear. Moreover, beyond perceptions of treatment, the extant research has yet to investigate the relationships between provider characteristics and other implementation outcomes. The present study seeks to advance research on these mixed findings by evaluating the relationships between provider characteristics and a range of adoption and implementation outcomes.

This study focuses on the implementation of an evidence-based psychological treatment (EBPTs). EBPTs target and reverse psychological processes that cause and/or maintain mental illness (Barlow et al., 2013; Chambless & Hollon, 1998). As such, EBPTs are widely regarded as frontline treatments for a range of serious mental illnesses (SMI) (Clark, 2018; American Psychological Association, 2006; National Institute for Health and Care Excellence, 2020). One promising treatment *target* for people with SMI is sleep and circadian problems, which cause impairment and predict and predate a range of SMI symptoms and diagnoses (e.g., major depressive disorder, psychosis, PTSD) (Dolsen et al., 2014; Hertenstein et al., 2019; Kivelä et al., 2018). Thus, the EBPT used in this study—Transdiagnostic Intervention for Sleep and Circadian Dysfunction (TSC^1^; Harvey & Buysse, 2017)—aims to target a range of sleep and circadian problems for individuals with a range of SMI diagnoses. TSC was delivered in a network of community mental health centers (CMHCs) which provide publicly funded mental health services in the United States, including to people with SMI.

The data for the present study were collected as part of a National Institute of Mental Health (NIMH) funded Hybrid Type 2 trial designed to evaluate the implementation and effectiveness outcomes of TSC in CMHCs (Harvey et al., 2024; Sarfan et al., 2023). For the secondary analysis presented herein, providers completed assessments at two timepoints: (1) immediately after they participated in the TSC training (i.e., provider post-*training* assessment) and (2) immediately after they delivered TSC to a patient (i.e., provider post-*treatment* assessment).

The first aim was to evaluate the extent to which provider characteristics were associated with provider perceptions of TSC prior to delivery, operationalized as provider ratings of treatment fit, and willingness and confidence to deliver TSC at the provider post-training assessment. We hypothesized that a higher education type and fewer years of experience would be associated with positive perceptions and that a higher case load would be associated with more negative perceptions. Additionally, we hypothesized that being licensed by the state of California (hereafter referred to as licensure status) and theoretical orientation (particularly CBT) would be associated with positive perceptions of TSC.

The second aim was to evaluate the extent to which provider characteristics, reported at the provider post-training assessment, were associated with other adoption and implementation outcomes, including (1) the number of patients the provider referred, (2) the average number of sessions a provider delivered, and (3) the number of patients who completed a course of treatment. The hypothesis tested is that a higher education type and fewer years of experience would be associated with a higher number of referrals, sessions, and completed courses of TSC and that a higher case load would be associated with fewer number of referrals, sessions, and completed courses of TSC. Additionally, we hypothesized that licensure status and theoretical orientation (particularly CBT) would be associated with a higher number of referrals, sessions, and completed courses of TSC.

The third aim was to evaluate the extent to which the implementation outcome of ratings of provider perceptions (i.e., treatment fit and willingness and confidence to deliver TSC), reported at the provider post-training assessment, predicted downstream the adoption and implementation outcomes, including (1) the number of patients the provider referred, (2) the average number of sessions a provider delivered, (3) the number of patients who completed a course of treatment, and (4) the rating of treatment fit at the provider post-treatment assessment. We hypothesized that providers who perceived the treatment to be a good fit and who reported higher willingness and confidence would have greater referrals, sessions, number of patients, and a higher rating of treatment fit at the provider post-treatment assessment.

## Method

Participants and data included in this study come from the aforementioned NIMH funded Hybrid Type 2 effectiveness-implementation cluster-randomized trial (R01MH120147). The parent trial consisted of three phases. The current study focuses on phase 1, the Implementation Phase, which evaluated the implementation and effectiveness outcomes of TSC across ten counties in California. See Sarfan et al. (2023) for full details on phase 1 of the parent trial. In an effort to improve the fit between TSC and the CMHC context, two versions of TSC were compared in the parent trial—Standard and Adapted TSC. Adapted TSC is a modified version of the Standard TSC modular approach, which distills the standard treatment from eight 50-minute sessions to four 20-minute sessions and compresses the content to the core building blocks commonly effective across a variety of sleep presentations.

### Participants

The inclusion criteria for selecting the CMHC sites within counties from which we recruited providers and patients were: (1) provision of publicly funded adult mental health outpatient services and (2) support from CMHC leadership.

Providers within these CMHCs were recruited, trained, and supervised by University of California, Berkeley (UCB) staff to deliver TSC. The inclusion criteria for providers were: (1) employed or able to deliver client-facing services to patients within the CMHC, (2) interested in learning and delivering TSC, and (3) volunteered to participate and formally consent to participate.

### Measures

The data were collected as part of the NIMH parent trial (R01MH120147). Only the measures analyzed for the aims of the present secondary analysis study are reported below. See Table 1 for the timing of each measure.

**Table 1:** Timeline of measures

Measures	Post-Training	Post-Treatment
Provider Characteristics	X	
AIM, IAM, FIM	X	X
Willingness and Confidence Scale	X	
Number of TSC Patients Referred, Sessions Delivered, and Completed Courses of Treatment		X

#### Provider Characteristics

Providers self-reported their demographic characteristics (i.e., sex, race, ethnicity) and professional characteristics, including their education (i.e., degree), years of experience in current/related roles, caseload, licensure status (i.e., state licensure), previous training in sleep treatment, number of supervision sessions attended, and theoretical orientation. Aligned with prior studies (Garcia et al., 2020; Nelson & Steele, 2008), theoretical orientation was classified as ‘CBT’ or ‘non-CBT’ for the purposes of the present analyses, with the latter category combining all other response options (i.e., client-centered, family systems, psychodynamic, humanistic, integrative/holistic). Based on prior research demonstrating that master’s-level providers are less likely than doctoral-level providers to receive training in sleep treatments (Gumport et al., 2023), education was categorized by degree type, with traditionally master’s-level degrees followed by doctoral-level degrees (i.e., marriage and family therapy, occupational therapy, social work, nursing, psychology, medical^2^, other). In this study, marriage and family therapy degree was used as the reference category, given its status as a master’s-level degree and that it possibly receives the least amount of sleep training (Novak & Gillis, 2022).

#### Acceptability of Intervention Measure (AIM), Intervention Appropriateness Measure (IAM), Feasibility of Intervention Measure (FIM)

The AIM, IAM, and FIM measure providers’ perceived fit of TSC. These self-report measures assess three implementation outcomes: acceptability, appropriateness, and feasibility of a given treatment (Weiner et al., 2017). Using a 5-point Likert scale (1 = *completely disagree*, 5 = *completely agree*), providers rated the three constructs with 12 items (4 items for each construct) (Weiner et al., 2017). The measures are scored by calculating the mean for each construct, with higher scores indicating greater acceptability, appropriateness, and feasibility. These measures have demonstrated satisfactory known-groups validity, internal reliability, test-retest reliability, and sensitivity to change (Weiner et al., 2017).

#### Willingness and Confidence Scale

Modeled after previous studies (Cunningham et al., 2018), providers self-report their willingness and confidence to deliver TSC with a single item for each construct. Providers were asked, “At this point, how willing are you to try out the sleep coaching?” and “At this point, how confident are you to deliver the sleep coaching?” Each item was rated on a 5-point Likert scale (1 = *not willing/confident*, 5 = *very willing/confident*). For the present analyses, the two constructs were combined into a single scale by taking the average of the two responses. Higher scores indicate greater willingness and confidence.

#### Number of TSC Patients Referred, Sessions Delivered, and Completed Courses of Treatment

For each provider, the number of patients referred to receive TSC was summed. During the trial, UCB staff recorded the number of TSC patients referred. For each provider, the number of sessions delivered (per patient) was averaged into one total score, representing average number of sessions delivered per patient. Additionally, per provider, the number of patients who completed a course of TSC was summed. A completed course of treatment was operationalized as completing, at minimum, the suggested number of sessions for each treatment condition (i.e., 8 sessions for Standard TSC and 4 sessions for Adapted TSC). During the trial, providers completed weekly logs via an online HIPAA-compliant Qualtrics survey to report when sessions were completed. From this information, number of sessions delivered per patient was tabulated and completed courses of treatment was determined from the tabulated number of sessions a given patient received.

### Treatment

TSC is a psychosocial treatment that treats a range of sleep and circadian problems experienced by people diagnosed with a range of SMI. Standard TSC (Harvey & Buysse, 2017) is a modular treatment comprised of 4 core modules, 4 cross-cutting modules, and 7 optional modules. It typically consists of eight weekly, 50-minute sessions. In an effort to improve the fit between TSC and the CMHC context, we adapted TSC. We grounded the process for adapting in theory (e.g., Escoffery et al., 2018; Lee et al., 2008), data (Gumport et al., 2019), and stakeholder input (Armstrong et al., 2022; Gumport et al., 2022). The Replicating Effective Programs (REP) framework (Kilbourne et al., 2007) was used as the overarching guide for the adaptation process. Adapted TSC is comprised of 5 core modules^3^, 4 cross-cutting modules, and 1 optional module. It typically consists of four weekly, 20-minute sessions. See Table 2 for treatment modules across conditions. In the analyses, treatment groups are combined to maximize the available provider characteristics data to assess impact on adoption and implementation outcomes. Lastly, to improve the fit between TSC and the CMHC context, reduce therapy-related stigma, and increase treatment approachability, both Standard and Adapted TSC were presented to patients in plain language (Larsen et al., 2025) as “Sleep Coaching,” while providers were referred to as “Sleep Coaches” or “Sleep Therapists.”

**Table 2:** Treatment modules across standard and adapted TSC condition

Cross Cutting				Standard TSC	Adapted TSC
Formulation	Education	Motivational Enhancement	Goal Setting	Core 1a: Regular Sleep Wake Times	Core 1: Regular Sleep-Wake Times
				Core 1b: Wind-down Routine	Core 2: Wind-down Routine
				Core 1c: Wake-up Routine	Core 3: Wake-up Routine
				Core 2: Improving Daytime Functioning	Core 4: Improving Daytime Functioning
				Core 3: Unhelpful Beliefs about Sleep	Core 5: Maintaining your Gains
				Core 4: Maintaining your Gains	Optional 1: Reducing Sleep Related Worry
				Optional 1: Improving Sleep Efficiency	
				Optional 2: Reducing Time in Bed	
				Optional 3: Delayed or Advanced Phase	
				Optional 4: Reducing Sleep-Related Worry	
				Optional 5: CPAP Machine and Exposure	
				Optional 6: Negotiating Complicated Environments	
				Optional 7: Reducing Nightmares	

### Procedure

Providers were consented by the trained UCB assessment team prior to participation. Providers attended a 4–8-hour TSC training either in-person or virtually (see Sarfan et al., 2023, for details). Providers were then encouraged to refer patients to the UCB staff, who determined if the patient was eligible. If the patient was eligible (see Supplement for patient inclusion criteria), the provider would deliver TSC to the patient. All providers delivering TSC were encouraged to attend supervision led by UCB staff. Provider assessments were completed immediately after participating in the TSC training (i.e., provider post-*training* assessment) and immediately after delivering TSC to a patient (i.e., provider post-*treatment* assessment). Providers also completed weekly logs to report when sessions were completed. At the provider post-training assessment, providers completed the measure of provider characteristics, AIM, IAM, FIM, and the Willingness and Confidence Scale. At the provider post-treatment assessment, providers completed the AIM, IAM, and FIM. Providers completed all assessments and weekly logs via online HIPAA-compliant Qualtrics surveys. Providers were compensated for completion of assessments, if permitted by their CMHC. The number of TSC patients referred were recorded by UCB staff during the trial.

### Allocation

CMHCs and patients were randomized through a computerized randomization sequence. CMHCs (including providers and patients) were cluster-randomized to Adapted vs. Standard TSC. Patients were randomized to immediate or delayed TSC. CMHCs were not stratified during randomization. Only the research team (i.e., not CMHCs, providers, or patients) were privy to which CMHCs were allocated to which TSC treatment condition (i.e., Adapted vs. Standard TSC). CMHC providers and patients knew whether the patient had been randomized to receive immediate or delayed treatment. In the immediate condition, the providers were asked to begin sessions as soon as possible. In the delayed condition, the providers were asked to wait until after the patient had completed the delay period (i.e., approximately 4 weeks in the Adapted condition or 8 weeks in the Standard condition). To maximize the sample size and because prior research has shown no significant difference between the treatment conditions (i.e., Adapted vs. Standard TSC) on ratings of fit (Diaz et al., in revision; Harvey et al., 2024), we collapsed over Standard and Adapted TSC providers in this study. Additionally, providers randomized to the delayed treatment group were included in the present secondary analyses. Of note, the number of providers and patients in this study differs from the main report (Harvey et al., 2024) due to the inclusion criteria: only provider-patient dyads in which the provider was enrolled (i.e., consented and provided treatment to at least 1 patient) were included, resulting in the exclusion of one patient (who did not complete treatment), additionally, providers from the delayed treatment group were included, leading to a larger provider sample compared to the main report (*N* = 93).

### Data Analysis

All analyses were conducted in R (version 4.3.1, R Foundation for Statistical Computing).

For Aim 1, four linear regression models were conducted to test whether CMHC providers’ characteristics, at post-training assessment, predicted (1) acceptability, appropriateness, and feasibility, and (2) willingness and confidence to deliver TSC, as measured at the post-training assessment. Each model included all providers’ characteristics (i.e., education, years of experience, caseload, licensure status, and theoretical orientation) as the predictors and one outcome (i.e., acceptability, appropriateness, feasibility, and willingness and confidence). All outcomes were modeled as continuous variables. Predictor variables (i.e., providers’ characteristics) are described below.

For Aim 2, three linear regression models were used to test whether CMHC providers’ characteristics, at post-training assessment, predicted (1) the number of patients the provider referred, (2) the average number of sessions a provider delivered (per patient), and (3) the number of patients who completed a course of treatment per provider. Each model included all providers’ characteristics (i.e., education, years of experience, caseload, licensure status, and theoretical orientation) as the predictors and one outcome (i.e., number of patients referred, average number of sessions delivered, and number of patients who completed a course of treatment). All outcomes were modeled as continuous outcome variables. Providers’ characteristics are described below.

For both Aims 1 and 2, CMHC providers’ characteristics, predictor variables, were included as follows: a dummy-coded variable for education (1 = *marriage and family therapy*; 2 = *occupational therapy;* 3 = *social work;* 4 = *nursing; 5 = psychology*; 6 = *medical*; 7 = *other*), years of experience and case load as continuous variables, a dummy-coded variable for licensure status (0 = *not licensed*, 1 = *licensed*), and a dummy-coded variable for theoretical orientation (0 = *non-CBT*, 1 = *CBT*). R^2^ is reported as an effect size and can be interpreted as the proportion of variance explained by the linear regression models. If a significant association is found for any education type, pairwise comparisons will be performed using Tukey’s test to correct for multiple comparisons and identify which group pairs differed significantly.

For Aim 3, three linear regression models and three mixed effect models were used to test whether rating of treatment fit and willingness and confidence to deliver TSC predict (1) number of patients the provider referred, (2) average number of sessions a provider delivered (per patient), (3) number of patients who completed a course of treatment, and (4) the rating of treatment fit at the provider post-treatment assessment. Each linear regression model included all ratings of treatment fit and willingness and confidence (i.e., acceptability, appropriateness, feasibility, and willingness and confidence) at post-training assessment as the predictor and one outcome (i.e., number of patients referred, average number of sessions delivered, number of patients who completed a course of treatment). All predictors and outcomes were modeled as continuous variables. Each mixed effects model included all ratings of treatment fit and willingness and confidence (i.e., acceptability, appropriateness, feasibility, and willingness and confidence) at post-training assessment as the predictor and one outcome (i.e., acceptability, appropriateness, and feasibility at provider post-treatment assessment). The random component of the model included a provider-specific random intercept to account for correlations between responses for a given provider. The standardized coefficients (β) can be interpreted as the change measured in units of a given outcome measure’s standard deviation. Standardized coefficients were obtained by fitting the model on a standardized version of the dataset. Standardized coefficients are reported as the effect size for mixed effects models.

#### Missing Data

The percentage of missing data ranged from 0% to 35.4% across variables and assessment timepoints. The pattern of missing data was assumed to be missing at random (MAR). Missing data were handled with multiple imputation (for linear regressions models) and maximum likelihood estimation (for mixed effects models), depending on the capabilities of the R packages used for each analysis. All analyses used the intention-to-treat population. Multiple imputation and maximum likelihood estimation both perform well with missing data that are MAR (Enders, 2017; Schafer & Graham, 2002) and perform well in simulations with missing data up to 50% (Black et al., 2011). Multiple imputation was conducted using the mice package (van Buuren & Groothuis-Oudshoorn, 2011).

## Results

Demographic characteristics of providers (*N* = 124) and patients (*N* = 395) can be found in Table 3. Descriptive statistics for outcome variables can be found in Table 4.

**Table 3:** Demographic characteristics of providers and patients

	Providers (*N* = 124)		Patients (*N* = 395)	
	*n*	%	*n*	%
Sex				
Male	11	9	150	38
Female	95	77	242	61
Non-binary	0	0	--	
Prefer not to say	2	2	3	1
Not specified	16	13	--	
Ethnicity				
Hispanic or Latino	35	28	141	36
Not Hispanic or Latino	63	51	249	63
Not specified	26	21	5	1
Race				
Native Hawaiian or Pacific Islander	2	2	8	2
American Indian or Alaskan Native	3	2	40	10
Asian	14	11	36	9
White	68	55	210	53
Black or African American	5	4	37	9
More than 1 race	7	6	57	14
Not specified	25	20	7	2
Licensure				
Licensed	65	52	--	
Not Licensed	41	33	--	
Not specified	18	15	--	
Degree				
Marriage and Family Therapy	34	27	--	
Psychology	13	10	--	
Social Work	37	30	--	
Nursing	1	1	--	
Medical	2	2	--	
Occupational Therapy	6	5	--	
Other	11	9	--	
Not specified	20	16		
Theoretical Orientation^a^				
Client-centered	77	62	--	
Family systems	29	23	--	
Cognitive Behavioral Therapy	70	56	--	
Psychodynamic	26	21	--	
Humanistic	3	2	--	
Integrative/Holistic	4	3	--	
None	4	3	--	
Not specified	22	18	--	
Prior Sleep Training			--	
Prior sleep training	9	7	--	
No prior sleep training	55	44	--	
Not specified	60	48	--	
Total Experience	*M* = 7.58	*SD* = 7.18	--	
Caseload	*M* = 33.4	*SD* = 28.42	--	
Supervision Attendance	*M* = 5.95	*SD* = 5.67	--	

^a^Providers could select multiple response options

**Table 4:** Means and standard deviations of outcome variables per provider across timepoints

	Post-Training			Post-Treatment		
Variables	*M*	SD		M	*SD*	
Acceptability	4.66	0.56		4.70	0.51	
Appropriateness	4.64	0.59		4.62	0.55	
Feasibility	4.56	0.59		4.65	0.54	
Willingness and Confidence	4.40	0.50		--	--	
Number of Patients Referred	--	--		3.23	3.10	
Average Number of Sessions	--	--		4.00	2.73	
Number of Patients Completed Treatment	--	--		1.51	1.75	

### Aim 1: Provider Characteristics and Provider Perceptions of TSC

Table 5 provides the coefficients for the linear regression models testing the relationship between provider characteristics and provider perceptions of TSC prior to delivery (i.e., acceptability, appropriateness, feasibility, and willingness & confidence at the provider post-training assessment). There were no significant findings (*p*s > .05), with *R*^2^ ≤ 0.11. Provider mean rating of the acceptability, appropriateness, and feasibility of TSC at post-training was at or above 4.56 on a 1–5 scale and the mean rating of willingness and confidence to deliver TSC was 4.40 on the same scale (see Table 4).

**Table 5:** Effect of provider characteristics on provider perceptions of TSC at Post-Training

	Acceptability											Appropriateness											Feasibility											Willingness & Confidence										
Predictor	*b*			SE		95% CI		*p*-value				*b*			SE		95% CI		*p*-value				*b*			SE		95% CI		*p*-value				*b*			SE		95% CI		*p*-value			
Occupational Therapy	0.41			0.24		−0.07, 0.89		.091				0.39			0.25		−0.11, 0.90		.124				0.32			0.26		−0.20, 0.85		.222				0.21			0.22		−0.23, 0.64		.349			
Social Work	0.00			0.13		−0.26, 0.26		.996				0.00			0.14		−0.27, 0.27		.994				−0.01			0.13		−0.28, 0.26		.938				0.14			0.12		−0.09, 0.37		.232			
Nursing	0.02			0.70		−1.36, 1.40		.982				−0.15			0.73		−1.60, 1.30		.838				−0.52			0.74		−2.00, 0.95		.481				0.86			0.61		−0.34, 2.07		.159			
Psychology	0.03			0.18		−0.33, 0.38		.873				0.13			0.18		−0.23, 0.50		.481				0.07			0.19		−0.30, 0.44		.701				−0.08			0.16		−0.39, 0.23		.616			
Medical	−0.24			0.41		−1.04, 0.57		.559				−0.43			0.42		−1.27, 0.41		.313				−0.47			0.42		−1.31, 0.37		.269				−0.68			0.36		−1.40, 0.03		.059			
Other degree	−0.05			0.20		−0.46, 0.35		.797				−0.15			0.22		−0.58, 0.28		.490				−0.10			0.22		−0.54, 0.34		.638				0.23			0.18		−0.13, 0.59		.213			
Total Experience	0.00			0.01		−0.02, 0.01		.665				0.00			0.01		−0.02, 0.02		.971				0.00			0.01		−0.02, 0.01		.814				0.00			0.01		−0.02, 0.01		.521			
Caseload	0.00			0.00		−0.00, 0.01		.464				0.00			0.00		−0.00, 0.01		.314				0.01			0.00		−0.00, 0.01		.059				0.00			0.00		−0.01, 0.00		.528			
Licensed	−0.08			0.12		−0.33, 0.16		.502				−0.12			0.13		−0.37, 0.14		.365				−0.07			0.13		−0.32, 0.19		.609				0.13			0.11		−0.09, 0.36		.233			
CBT	0.03			0.12		−0.22, 0.27		.819				0.12			0.13		−0.13, 0.37		.340				−0.01			0.13		−0.26, 0.24		.952				0.11			0.11		−0.11, 0.32		.323			
*R* ^2^								0.05											0.08											0.09											0.11			

**Table 6:** Effect of provider characteristics on adoption and implementation outcomes at post-treatment

	Number of Patients Referred						Average Number of Sessions						Number of Patients Completed Treatment					
Predictor	*b*		SE	95% CI	*p*-value		*b*		SE	95% CI	*p*-value		*b*		SE	95% CI	*p*-value	
Occupational therapy	**4.02**		**1.29**	**1.47**,** 6.58**	**.002**		0.46		1.29	−2.10, 3.03	.721		**2.49**		**0.80**	**0.88**,** 4.10**	**.003**	
Social work	0.07		0.68	−1.28, 1.42	.915		0.52		0.64	−0.74, 1.79	.414		0.05		0.38	−0.72, 0.82	.899	
Nursing	−3.21		4.40	−12.07, 5.65	.469		−2.12		3.52	−9.11, 4.87	.548		2.71		2.42	−2.17, 7.58	.269	
Psychology	**2.55**		**0.95**	**0.66**,** 4.44**	**.009**		0.11		0.88	−1.64, 1.86	.902		0.61		0.54	−0.48, 1.69	.269	
Medical	−0.49		2.28	−5.08, 4.04	.831		−3.17		1.97	−7.08, 0.74	.111		−1.49		1.17	−3.82, 0.83	.204	
Other degree	0.63		1.17	−1.71, 2.96	.593		−0.10		1.01	−2.12, 1.91	.918		−0.23		0.60	−1.42, 0.95	.698	
Total experience	0.05		0.04	−0.03, 0.13	.249		0.01		0.04	−0.06, 0.09	.710		0.02		0.02	−0.02, 0.07	.294	
Caseload	0.03		0.02	−0.01, 0.07	.182		0.01		0.01	−0.02, 0.04	.529		−0.01		0.01	−0.03, 0.02	.575	
Licensed	−0.16		0.65	−1.45, 1.13	.807		1.18		0.62	−0.06, 2.42	.062		0.47		0.37	−0.26, 1.20	.205	
CBT	0.61		0.64	−0.65, 1.88	.339		0.41		0.59	−0.77, 1.59	.492		0.24		0.37	−0.49, 0.98	.509	
*R* ^2^					0.19						0.11						0.26	

### Aim 2: Provider Characteristics and Adoption and Implementation Outcomes

Table 6 provides the coefficients for the linear regression models testing the association between provider characteristics and adoption and implementation outcomes (i.e., number of patients referred, average number of sessions delivered, and number of patients who completed a course of treatment). For number of patients referred, providers with degrees in occupational therapy (*b* = 4.02, *p* =.002) and psychology (*b* = 2.55, *p* =.009) referred higher number of patients, relative to the reference degree (i.e., marriage and family therapy). On average, occupational therapy providers referred 7 patients and psychology providers 5, compared to 3 among marriage and family therapy providers. The model explained a moderate portion of the variance in outcome variables, *R*^2^ = 0.19. Post hoc pairwise comparisons using tukey’s test indicated that occupational therapists referred a significantly higher number of patients relative to marriage and family therapists (*p* =.037) and social workers (*p* =.046). No other comparisons survived correction for multiple comparisons. For average number of sessions delivered^4^, no significant associations were identified. For number of patients who completed a course of treatment, one category of degree was statistically significant, occupational therapy (*b* = 2.49, *p* =.003), indicating that occupational therapists treated a higher number of patients who completed a course of treatment, relative to the reference degree (i.e., marriage and family therapy). On average, occupational therapy providers completed a course of treatment with 5 patients, compared to 1 among marriage and family therapy providers. The model explained a moderate-to-large portion of the variance in outcome variables, *R*^2^ = 0.26. Post hoc pairwise comparisons using tukey’s test indicated that occupational therapists completed treatment with a significantly higher number of patients relative to marriage and family therapists (*p* =.046) and approached significance when compared to social workers (*p* =.058) and medical professionals (*p* =.057). No other comparisons survived correction for multiple comparisons. All significant models explained a moderate portion of the variance in outcome variables (*R*² ≥ 0.19), which corresponds to a medium effect size

### Aim 3: Provider Perceptions of TSC and Adoption and Implementation Outcomes

Table 7 provides the coefficients for the linear regression models and the standardized coefficients (β) for the mixed effects models testing the association between provider characteristics and adoption and implementation outcomes (i.e., number of patients referred, average number of sessions delivered, number of patients who completed a course of treatment, and the ratings of treatment fit at the provider post-treatment assessment). There were no statistically significant findings between provider perceptions of TSC and the number of patients referred, average number of sessions delivered, or number of patients who completed a course of treatment. However, significant associations did emerge between provider perceptions of TSC from post-training to post-treatment. Specifically, providers’ ratings of appropriateness at post-training were significantly associated with all post-treatment ratings of fit: acceptability (*β* = 0.46, *p* =.006), appropriateness (*β* = 0.36, *p* =.039), and feasibility (*β* = 0.33, *p* =.052). These findings suggest that higher appropriateness ratings at post-training were linked to higher ratings of acceptability, appropriateness, and feasibility at post-treatment. The standardized coefficients suggest medium-to-large effects for acceptability and medium effects for appropriateness and feasibility. Additionally, providers’ ratings of feasibility at post-training was significantly associated with acceptability at post-treatment (*β* = −0.38, *p* =.004), such that higher feasibility ratings at post-training were linked to lower acceptability rating at post-treatment. This standardized coefficient suggests a medium effect size. Thus, the standardized coefficients in all significant models suggested at least a medium effect size. Lastly, provider mean ratings of the acceptability, appropriateness, and feasibility of TSC at post-treatment were above 4.62 on a 1–5 scale (see Table 4).

**Table 7:** Effect of provider perceptions of TSC at post-training on adoption and implementation outcomes at post-treatment

			Number of Patients Referred				Average Number of Sessions						Number of Patients Completed Treatment					
Predictor	*b*		SE	95% CI	*p*-value		*b*		SE	95% CI	*p*-value		*b*		SE	95% CI	*p*-value	
Acceptability	0.00		1.11	−2.19, 2.19	.999		−1.29		1.05	−3.38, 0.80	.222		−0.17		0.66	−1.50, 1.16	.795	
Appropriateness	0.66		1.02	−1.35, 2.68	.517		1.05		0.94	−0.81, 2.92	.266		0.70		0.59	−0.51, 1.85	.241	
Feasibility	0.31		0.87	−1.42, 2.04	.724		−0.22		0.79	−1.79. 1.35	.782		−0.21		0.54	−1.17, 0.82	.705	
Willingness & Confidence	−0.13		0.64	−1.40, 1.14	.840		−0.14		0.59	−1.30, 1.02	.811		0.31		0.37	−0.43, 1.04	.409	
*R* ^2^					0.03						0.03						0.04	
			Acceptability at Post-Treatment				Appropriateness at Post-Treatment				Feasibility at Post-Treatment							
Predictor	*β*		SE	95% CI	*p*-value		*β*		SE	95% CI	*p*-value		*β*		SE	95% CI	*p*-value	
Acceptability	0.04		0.16	−0.27, 0.36	.820		−0.09		0.17	−0.43, 0.25	.590		−0.19		0.16	−0.51, 0.15	.259	
Appropriateness	**0.46**		**0.16**	**0.13**,** 0.77**	**.006**		**0.36**		**0.17**	**0.02**,** 0.70**	**.039**		**0.33**		**0.17**	**−0.00**,** 0.66**	**.052**	
Feasibility	**−0.38**		**0.13**	**−0.63**, **−0.13**	**.004**		−0.14		0.14	−0.42, 0.12	.291		−0.02		0.13	−0.28, 0.24	.888	
Willingness & Confidence	0.14		0.08	−0.03, 0.30	.097		0.13		0.09	−0.04, 0.31	.140		0.09		0.09	−0.08, 0.27	.279	

## Discussion

Dissemination and implementation frameworks, such as EPIS, CFIR and RE-AIM, highlight the influence of provider characteristics on adoption and implementation outcomes. Despite this, the impact of these factors remains understudied. The present study evaluates how provider characteristics and providers’ perceptions of TSC are related to each other and how they influence adoption and implementation outcomes within CMHCs. The insights gained from this research are intended to support community organizations and researchers in making informed decisions regarding the recruitment of providers and the evaluation of adoption and implementation outcomes when implementing sleep and circadian EBPTs in CMHCs. They also contribute to the limited evidence on which provider characteristics may—or may not—play an important role in adoption and implementation outcomes.

Aim 1 examined the relationship between provider characteristics and their perceptions of TSC—specifically, their perceived acceptability, appropriateness and feasibility of TSC, along with their willingness and confidence to deliver TSC. This was assessed at the provider post-training assessment, prior to TSC delivery. Consistent with mixed findings reported in the literature regarding provider characteristics such as education, experience, licensure, and theoretical orientation, no statistically significant associations were found between these provider characteristics and their perceptions of TSC. With regard to caseload, although prior research has associated higher caseloads to more negative perceptions of interventions (e.g., Barnett et al., 2017; Brennan et al., 2022; Nelson et al., 2006), the current study did not identify a statistically significant relationship. Despite these non-significant associations, mean ratings for all provider perceptions of TSC fit and willingness and confidence at post-training were notably high. Providers rated the acceptability, appropriateness, and feasibility of TSC above 4.66 on a 1–5 scale, indicating that providers found TSC to have a strong fit. Willingness and confidence to deliver TSC also received a high mean rating of 4.44 on the same scale. These findings suggest that TSC was perceived favorably by providers across a range of characteristics and backgrounds. This may indicate that TSC is particularly well-suited for CMHCs, where provider characteristics are diverse and treatment delivery is not limited to a specific professional role (e.g., licensed clinical psychologist).

Aim 2 evaluated how provider characteristics at the post-training assessment (i.e., prior to TSC delivery) relate to adoption and implementation outcomes, including the number of patients the provider referred, the average number of sessions a provider delivered, and the number of patients who completed treatment. In partial support of the hypothesis, education type was significantly associated with select adoption and implementation outcomes, with *R*^2^ indicating a medium effect size. Specifically, providers with an occupational therapy degree referred more patients and had higher treatment completion rates, while providers with a psychology degree also referred more patients. However, for psychology degree the findings should be interpreted cautiously, as statistical significance was not retained after correction for multiple comparisons. That said, further research is needed to understand why occupational therapists and psychologists were more likely to adopt and implement TSC. One possibility is that sleep education and training may differ across degrees and professions. For example, sleep and rest are core components of the occupational therapy framework (Boop et al., 2020), potentially resulting in more extensive training on sleep. Similarly, psychologists have reported a median of 10 h of sleep training (Zhou et al., 2021). While limited, this suggests at least some exposure to sleep education. In contrast, some findings suggest that sleep education is rarely included in training for social work and marriage and family therapy programs (Novak & Gillis, 2022; Spadola et al., 2024). Additionally, occupational therapists and psychologists may be more likely to adopt and implement sleep interventions because such interventions align closely with the scope of their professional roles (Boop et al., 2020; Zhou et al., 2021). Lastly, the other provider characteristics—namely, experience, caseload, licensure, and theoretical orientation—were not significantly associated with any adoption and implementation outcomes. To the best of our knowledge, these variables have shown mixed associations with treatment perceptions and have not been examined directly in relation to adoption and implementation outcomes. We hope that the present investigation encourages the inclusion of these variables in future implementation research. Together, these findings further support and extend the EPIS, CFIR, and RE-AIM frameworks (Aarons et al., 2011; Damschroder et al., 2022; Glasgow et al., 1999) by underscoring the importance of assessing not only how provider characteristics influence general attitudes toward adopting EBPs, but also how they impact actual adoption and implementation outcomes.

Aim 3 examined how provider perceptions of TSC (i.e., treatment fit and willingness and confidence to deliver TSC) at the provider post-training assessment (i.e., prior to TSC delivery), predict adoption and implementation outcomes (i.e., number of patients the provider referred, average number of sessions a provider delivered, number of patients who completed a course of treatment, and the rating of treatment fit) at the provider post-treatment assessment. Findings partially supported our hypotheses, aligning with prior research suggesting that holding a positive perception of interventions has been associated with downstream adoption and implementation outcomes (e.g., Gallo & Barlow, 2012; Nelson & Steele, 2007). In the present study, providers’ post-training ratings of the appropriateness of TSC were positively associated with provider ratings of the acceptability, appropriateness, and feasibility of TSC at post-treatment, with standardized coefficients suggesting a medium effect size. Additionally, providers’ post-training rating of the feasibility of TSC were negatively associated with provider ratings of acceptability of TSC at post-treatment, with standardized coefficients also suggesting a medium effect size. It is possible that, although providers initially perceived TSC as feasible at post-training, they may have later felt that it was not sufficiently in-depth or useful following its implementation. No other significant associations were found for the other adoption and implementation outcomes. Importantly, treatment providers’ perceptions of TSC (i.e., treatment fit) remain high at post-treatment, as indicated by mean scores above 4.62 on the 1–5 scale, indicating strong acceptability, appropriateness, and feasibility of TSC. In sum, providers’ perceptions of TSC following training but prior to the delivery of TSC largely remained steady following adoption and implementation, underscoring TSC’s strong fit within the CMHC setting.

Several limitations should be considered when interpreting the present findings. First, the assessment of outcomes relied on provider self-report, which may be subject to bias. Second, we did not collect data from or assess providers who were trained but did not participate in the study or patients referred who were ineligible for the study. As highlighted in the RE-AIM framework (Glasgow et al., 1999), to fully understand adoption and implementation outcomes, future research should track the characteristics of non-participants, for example, the number of those trained or asked to participate, perceptions of providers who did not participate in the study, or the number of patients referred to treatment but did not consent to participate. Third, this study focused on providers delivering a single treatment (TSC) within one context (CMHCs). Investigating the generalizability of these findings to other populations and settings (e.g., VA or academic-medical settings) is an important area for future research. Fourth, while we collected data on providers’ degree type, we did not systematically collect data on their education level (i.e., master’s versus doctorate).

In conclusion, the present study enhances our understanding of how provider characteristics and perceptions influence adoption and implementation outcomes of sleep and circadian interventions. We found that, CMHC provider characteristics (i.e., education, experience, caseload, licensure, and theoretical orientation) did not influence providers perceptions of TSC’s fit or willingness and confidence to deliver the treatment. However, CMHC providers’ perceptions of TSC were generally highly positive. Furthermore, with the exception of education type, most provider characteristics did not appear to influence adoption and implementation outcomes (i.e., number of patients referred, average number of sessions delivered, number of patients who completed treatment). Additional research is needed to more clearly understand how provider characteristics, such as education and training, may influence adoption and implementation outcomes. Finally, early perceptions of the treatment’s appropriateness and feasibility appear to influence later perceptions of treatment fit.

## Data Availability

The data that support the findings of this study are available from the corresponding author upon reasonable request.

## Footnotes

1 Previously referred to as TranS-C.

2 Psychology and medical providers were primarily trained at the doctorate level. Furthermore, nursing included some providers with doctoral degrees.

3 Adapted TSC consists of the same core modules as Standard TSC, except that Unhelpful Beliefs about Sleep is no longer included. This is a clarification of our protocol papers for this study (Callaway et al., 2023; Sarfan et al., 2023).

4 CMHC providers sometimes delivered more sessions than recommended due to unique factors and complications that lead to longer treatment durations as the norm in CMHC settings (e.g., treatment pauses, canceled or rescheduled sessions, competing health priorities or crises), clinical needs (e.g., comorbidity, complex sleep issues), and/or provider or patient preferences (e.g., interest in sleep and a desire to learn more).
